# Multi-Agent Reinforcement Learning Based Fully Decentralized Dynamic Time Division Configuration for 5G and B5G Network

**DOI:** 10.3390/s22051746

**Published:** 2022-02-23

**Authors:** Xiangyu Chen, Gang Chuai, Weidong Gao

**Affiliations:** Department of Information and Communication Engineering, Beijing University of Posts and Telecommunications, Beijing 100876, China; xychen324@bupt.edu.cn (X.C.); gaoweidong@bupt.edu.cn (W.G.)

**Keywords:** dynamic TDD, MARL, leniency control, 5G and B5G, decentralized network

## Abstract

Future network services must adapt to the highly dynamic uplink and downlink traffic. To fulfill this requirement, the 3rd Generation Partnership Project (3GPP) proposed dynamic time division duplex (D-TDD) technology in Long Term Evolution (LTE) Release 11. Afterward, the 3GPP RAN#86 meeting clarified that 5G NR needs to support dynamic adjustment of the duplex pattern (transmission direction) in the time domain. Although 5G NR provides a more flexible duplex pattern, how to configure an effective duplex pattern according to services traffic is still an open research area. In this research, we propose a distributed multi-agent deep reinforcement learning (MARL) based decentralized D-TDD configuration method. First, we model a D-TDD configuration problem as a dynamic programming problem. Given the buffer length of all UE, we model the D-TDD configuration policy as a conditional probability distribution. Our goal is to find a D-TDD configuration policy that maximizes the expected discount return of all UE’s sum rates. Second, in order to reduce signaling overhead, we design a fully decentralized solution with distributed MARL technology. Each agent in MARL makes decisions only based on local observations. We regard each base station (BS) as an agent, and each agent configures uplink and downlink time slot ratio according to length of intra-BS user (UE) queue buffer. Third, in order to solve the problem of overall system revenue caused by the lack of global information in MARL, we apply leniency control and binary LSTM (BLSTM) based auto-encoder. Leniency controller effectively controls Q-value estimation process in MARL according to Q-value and current network conditions, and auto-encoder makes up for the defect that leniency control cannot handle complex environments and high-dimensional data. Through the parallel distributed training, the global D-TDD policy is obtained. This method deploys the MARL algorithm on the Mobile Edge Computing (MEC) server of each BS and uses the storage and computing capabilities of the server for distributed training. The simulation results show that the proposed distributed MARL converges stably in various environments, and performs better than distributed deep reinforcement algorithm.

## 1. Introduction

Mobile data traffic is forecasted to grow significantly because of the rapid change in patterns of application services and massive explosion in use of connected devices. On the other hand, 5G and B5G (beyond 5G) network needs to provide UEs with services in different scenarios, such as URLLC, IoT, and IoV [1]. The behavior of UEs in these scenarios is also different. This is to say, the volume and pattern of network traffic will change rapidly. In response to this sudden surge, D-TDD is chosen as a possible solution [2,3]. The traditional static TDD (S-TDD) synchronizes the uplink/downlink (UL/DL) slots ratio configuration of all BSs. However, D-TDD technology dynamic changes the ratio of downlink/uplink slots for traffic adaptation. This bring two gains to the system [4]:

(1) High Time Resource Utilization: Since S-TDD fix ratio of UL/DL, the probability that the ratio does not fit traffic pattern increases. This causes the situation, for example, where no data are transmitted in the downlink time slot because the ratio of downlink slots is less than ratio of downlink services. In contrast, D-TDD dynamic configures UL/DL slots ratio, which improve time resource utilization [5,6].

(2) Low Latency: D-TDD can also reduce latency [7]. This is because dynamic ratio of UL/DL configuration reduces the queue length of buffer.

However, flexible UL/DL ratio configuration can future result in cross-link interface (CLI) [8]. Compared with inter-BS interference, CLI has a greater impact on uplink transmission. This is caused by unequal uplink and downlink transmission power. Due to hardware limitations, transmit power of UE’s mobile device is usually less than transmit power of BS. Therefore, the interference power generated by the downlink transmission of neighboring BSs seriously affect the quality of uplink transmission service. On the other hand, 5G NR provides more flexible duplex pattern for D-TDD, but it still lacks a D-TDD configuration scheme [9]. So the adaptive dynamic TDD configuration scheme becomes an important research subject. Article [10] considers UE association and D-TDD configuration, then proposes a joint solution. Article [11] proposes successful approximation of fixed point (SAFP) and resource muting for dominant interference (RMDI) algorithm to solve the D-TDD configuration problem.

The above existing work on D-TDD generally uses a centralized solution, which has two shortcomings: signaling overhead and control delay. First, the centralized solution needs to be deployed on a centralized controller or cloud servers. The centralized solution requires transmission of data from end devices to a centralized controller or cloud server [12], which leads to high transmission, computing and signaling costs [13]. Secondly, the transmission of data and the issuance of control commands in centralized solution cause additional delays. Coupled with the calculation time of the algorithm, many scenarios and services, such as URLLC [14], cannot accept the high time cost of a centralized solution. Moreover, 5G and B5G network topology is more complex and provides different types of services to UE, which puts forward higher requirements to D-TDD technology. According to the paper [15], 5G and B5G network should be concise and expandable, and centralized solutions reduce the expandability of network.

Coping with this issue, a distributed solution becomes an option. In contrast with centralized schemes, distributed solutions can improve computation efficiency by parallel execution at distributed locations. Considering that the wireless communication network is a natural distributed system, a decentralized solution can be distributed deployed in each BS. Combined with MEC technology, data collection and related computing tasks are performed by MEC servers. This reduces the control delay and signaling overhead. In the paper [16], the system-level simulation of centralized and decentralized dynamic TDD resource allocation schemes were conducted. The results show that distributed D-TDD improves resource utilization and reduces latency.

In recent years, MARL, as a new pattern of the decentralized solution, has attracted the attention of many researchers [17]. MARL expends reinforcement learning to the field of the multi-agent environment. The task of learning in a multi-agent environment is more complicated than that in a single-agent environment, because agent interacts with environment and other agents at the same time, which increases the non-stationarity of the learning environment. In order to improve the performance of learning in a multi-agent environment, researchers generally use four methods: (1) Extended reinforcement learning: extend RL to multi-agent environment directly; (2) Learning communication: agent communicates with each other to complete learning task; (3) Learning cooperation: agent learns to cooperate without communication; (4) Agents modeling agents: agent reason about the behaviors of other agents by predicting other agents.

In addition to MARL, federated learning (FL) is also seen as a distributed learning solution. FL is a machine learning method in which distributed learning nodes (e.g., mobile devices) collaboratively train a model under the orchestration of a central controller (e.g., service provider), while keeping the training data decentralized [18]. According to paper [19], FL can be divided into three categories: (1) Horizontal Federated Learning: datasets in distributed nodes share the same feature space but are different in samples; (2) Vertical Federated Learning: datasets in distributed nodes differ in feature space but share the same feature space; (3) Federated Transfer Learning: datasets in distributed nodes are different in samples and feature space. Decentralized training data can mitigate the privacy and overhead issues of traditional centralized machine learning. Thus, some researchers give rise to the idea of federated reinforcement learning (FRL) which can be considered as an integration of FL and RL [20]. According to paper [21], MARL research is similar with FRL research [22], but FRL has advantages of high privacy protection and wide application scenarios. Therefore, the paper [23] proposed a federated RL-based channel resource allocation framework for 5G/B5G networks, and suggested collaborating learning estimates for faster learning convergence. In the paper, the authors deploy local learning models (LLMs) and global learning models (GLMs) in distributed nodes (UE) and centralized controllers (APs), respectively. Every UE integrates locally updated value in a federated ACK (FACK) message, which is fed to APs for training GLM.  UE trains LLM combining the feedback from the local exploration and the Q-value estimated by GLM.

FRL has advantages of high privacy protection and wide application scenarios, but to the best knowledge of the authors, almost all FRL solutions cause additional signaling and data transmission. This is because FRL based solutions require a centralized server to control the interaction of distributed nodes. Considering our motivation for designing a decentralized solution: avoiding additional signaling and data transmission. Learning cooperation is a suitable MARL method. Each agent makes policy decisions based on local observations without information of other agent. Furthermore, we need to solve two problems in learning cooperation: (1) The non-stationarity of agent learning environment [24] caused by lack of global state information; (2) Relative over-generalization: a sub-optimal policy in joint action space is preferred over an optimal policy for distributed agent [25]. Consider in the field of wireless communication, for example, each BS (seen as an agent) needs to configure UL/DL slots ratio (seen as an action) according to service buffer and channel conditions of the intra-BS UE (seen as local observations). For the lack of system configuration information, it is difficult for a BS to learn the impact of inter-BS interference on data rate (seen as a local reward) through exploration. In other words, from the perspective of distributed agents, the agent’s decision-making process no longer meets the Markov decision-making conditions [24]. This is the non-stationarity that distributed MARL encounters. On the other hand, each BS can only obtain transmission rate information itself, and cannot obtain the influence itself’s D-TDD configuration on the overall system rate (sum of each BS’s UL/DL transmission rate.). This leads to a BS making a resource allocation decision which is most beneficial to itself, thereby reducing the overall system rate. This is the relative over-generalization that MARL needs to face.

Even though several standard activities and related studies exist in the literature, providing efficient D-TDD configuration in 5G and B5G network still remains an open research area. In fact, the problem is complex and multifaceted, and, therefore, no single technology seems to be able to provide an effective solution. To the best of our knowledge, none of the existing studies proposed a fully decentralized solution to solve the problem of D-TDD configuration. Therefore, in this paper, we apply leniency control based MARL (LC-MARL) and propose a fully decentralized D-TDD configuration scheme to effectively accommodate different traffic demands of uplink/downlink service. Our contribution is:

- We formulate D-TDD configuration problem as a dynamic programming problem, and design a distributed MARL based decentralized solution;
- In order to obtain a higher overall system gain of distributed MARL, we apply a leniency control based learning cooperation method. Leniency controller reduces the influence of non-stationary learning environment on agent by effectively controlling the Q value estimation in MARL according exploration level and reward (buffer length under D-TDD configuration);
- BLSTM based binary auto-encoder is designed to extra the features of high dimensional time series and generate binary hashing-code, which increases the ability of leniency control;
- The proposed fully decentralized D-TDD configuration scheme requires no information interactions between BSs, which save the resource caused by transmission of control signaling and data.

## 2. Materials and Methods

### 2.1. System Model

#### 2.1.1. Network

**Figure 1:**
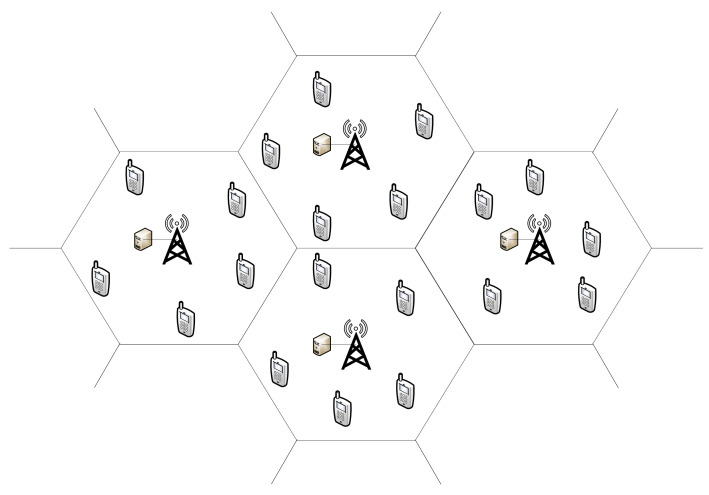
Wireless network.

Figure 1 illustrates a cellular wireless network, where each hexagon corresponds to one BS coverage area. UEs are distributed as a Poisson point process $\Phi_{UE}$ with density λ in each BS coverage area. We denoted UEs by $u\in U=\{1,2,\cdots ,N_{ue}\}$ which $N_{ue}$ is number of UEs. BS are denoted by $b\in B=\{1,2,\cdots ,N_{bs}\}$ which $N_{bs}$ is the number of BS. Each BS uses orthogonal frequency-division multiplexing (OFDM) transmission method to provide UEs with data transmission services. With  OFDM, UEs connect to the same BS are orthogonal with each other and no intra-BS interference exists. We assume that all BSs reuse the same frequency and bandwidth resources, which lead to inter-BS interference. Let $c\in C=\{1,2,\cdots ,N_{c}\}$ denote reused orthogonal sub-channels of each BS, with $N_{c}$ is the number of orthogonal sub-channels.

Thus, path loss of UE *u* between BS *b* on channel *c* is

(1) $$PL_{u,b}^{c}=32.4+21log_{10}\left(d_{u,b}\right)+20log_{10}\left(f_{c}\right)$$

where $d_{u,b}$ is the distance between UE *u* and BS *b*, and $f_{c}$ denote the frequency of sub-channel. We compute the channel gain at time t of the transmission between UE *u* and BS *b* over sub-channel *c* as follows:

(2) $$g_{u,b}^{c}=\left|h_{u,b}^{c}\right|PL_{u,b}^{c}$$

where $h_{u,b}^{c}$ is the small-scale fading component. Moreover, each UE selects BS which provides the max RSRP as service BS, and sets the value of $\eta_{u,b}$ to 1, which indicates the association between UE *u* and BS *b*.

#### 2.1.2. 5G NR Duplex Pattern

Time Division Long Term Evaluation (TD-LTE) fixes each radio frame to 10 slots, and the time length of each slot is 1 millisecond. In addition, uplink and downlink duplex pattern of TD-LTE is fixed. This means that the number of uplink and downlink time slots cannot be adjusted according to the service buffer situation. In order to improve the quality of service for UEs, 5G NR provides a variety of radio frame structure settings, and the uplink and downlink duplex pattern is more flexible.

The 5G defines a numerology parameter μ∈{0,1,2,3,4}. The 5G NR specifies five physical layer numerologies, which result in different sub-carrier spacing (SCS) and slot durations. For the sake of paper readability, we summarized the 5G NR supported transmission numerology and relevant information in Table 1. For the details, the SCS and slot duration are calculated by $15*2^{\mu }$ and $1/2^{\mu }$, respectively. The 5G NR still fixes the number of sub-frames in each radio frame and the number of symbols in each slot with 10 and 14. However, we can change the number of slots in a sub-frame which is calculated by $2^{\mu }$.

In the 5G NR protocol, BS informs UE of uplink/downlink pattern and time slot configuration through broadcast or RRC configuration message. The key parameter of uplink/downlink pattern is UL/DL transmission periodicity, denoted as δ. δ indicates the repeated period of UL/DL pattern. According to different Numerology parameter configuration μ, δ value has different options (see Table 2). For example, when μ=2, δ can only be set to 1.25 ms or 2.5 ms. Moreover, the 5G NR implements symbol-level time division duplex and sets time slots configuration through several parameters:

- $d_{slots}$: the number of downlink time slots after the start of transmission period.
- $u_{slots}$: the number of uplink time slots before the end of transmission period.
- $d_{sym}$: the number of downlink symbols in the time slot after the last complete downlink time slot.
- $u_{sym}$: the number of UL symbols in the end of a slot preceding the first full UL slot.

**Table 1:** Supported transmission numerologies and additional info.

μ	SCS	Number of Slots Per Frame	Time Length of Slot
0	15 khz	10	1 ms
1	30 khz	20	0.5 ms
2	60 khz	40	0.25 ms
3	120 khz	80	0.125 ms
4	240 khz	60	0.0625 ms

The remaining symbols are regarded as flexible symbols which can be allocated to uplink or downlink by dedicated configuration. So, we can obtain the transmission direction τ of BS *b* at time *t*. When $\tau_{b}\left(t\right)=1$, UE receives downlink data transmitted by BS, and when $\tau_{b}\left(t\right)=0$, UE sends uplink data to BS.

**Table 2:** Supported transmission numerologies and period of UL/DL pattern.

δ(ms)	μ	SCS (kHz)
0.625	3	120
1.25	2, 3	60, 120
2.5	1, 2, 3	30, 60, 120

#### 2.1.3. Problem Formulation

We consider a static power transmission scheme, where uplink transmission power of all UEs is the same, and downlink transmission power of all BSs is also the same. Uplink transmission power and downlink transmission power between UE *u* and BS *b* on each sub-channel *c* are denoted as $P_{u,b}^{ul}$ and $P_{u,b}^{dl}$, respectively. In order to ensure the fairness of UEs, we use a round-robin (RR) algorithm for sub-channel resource allocation. If sub-channel *c* is allocated to UE *u*, we set the indicator $\kappa_{u,b}^{c}$ to 1, otherwise 0. Therefore, the uplink and downlink transmission SINR of UE *u* at time *t* can be written as Equations (3) and (4).

(3) $$\gamma_{u}^{ul}\left(t\right)=\frac{\sum_{b\in B}\sum_{c\in C}\eta_{u,b}\cdot \kappa_{u,b}^{c}\cdot P_{u,b}^{ul}\cdot g_{u,b}^{c}}{\sigma^{2}+\sum_{u\in U}\sum_{b\in B}\sum_{c\in C}I_{u,b}^{ul}\left(t\right)+\sum_{u\in U}\sum_{b\in B}\sum_{c\in C}I_{u,b}^{dl}\left(t\right)}$$

(4) $$\gamma_{u}^{dl}\left(t\right)=\frac{\sum_{b\in B}\sum_{c\in C}\eta_{u,b}\cdot \kappa_{u,b}^{c}\cdot P_{u,b}^{dl}\cdot g_{u,b}^{c}}{\sigma^{2}+\sum_{u\in U}\sum_{b\in B}\sum_{c\in C}I_{u,b}^{ul}\left(t\right)+\sum_{u\in U}\sum_{b\in B}\sum_{c\in C}I_{u,b}^{dl}\left(t\right)}$$

(5) $$I_{u,b}^{ul}\left(t\right)=\left(1-\tau_{b}\left(t\right)\right)\eta_{u,b}\cdot \kappa_{u,b}^{c}\cdot P_{u,b}^{ul}\cdot g_{u,b}^{c}$$

(6) $$I_{u,b}^{dl}\left(t\right)=\tau_{b}\left(t\right)\eta_{u,b}\cdot \kappa_{u,b}^{c}\cdot P_{u,b}^{dl}\cdot g_{u,b}^{c}$$

where $\sigma^{2}$ is additive white Gaussian noise power spectral density, $I_{u,b}^{ul}\left(t\right)$ and $I_{u,b}^{dl}\left(t\right)$ are UL and DL interference, respectively. Furthermore, UL and DL transmission rate $r_{u}^{ul}\left(t\right)$ and $r_{u}^{dl}\left(t\right)$ of UE *u* are computed as follows:

(7) $$r_{ul}^{u}\left(t\right)=BW\cdot \log_{2}\left(1+\gamma_{u}^{ul}\right)$$

(8) $$r_{dl}^{u}\left(t\right)=BW\cdot \log_{2}\left(1+\gamma_{u}^{dl}\right)$$

where BW is the bandwidth of sub-channel. We assume $\varrho_{u}^{ul}\left(t\right)$ and $\varrho_{u}^{dl}\left(t\right)$ are the UL/DL packet size generated by UE *u* at time *t*, then we can obtain total uplink buffer length $\Omega^{ul}\left(t\right)$ and total downlink buffer length $\Omega^{dl}\left(t\right)$ by

(9) $$\Omega^{ul}\left(t\right)=\sum_{b\in B}\Omega_{b}^{ul}\left(t\right)=\sum_{b\in B}\sum_{u\in b}\omega_{u}^{ul}\left(t\right)=\sum_{b\in B}\sum_{u\in b}\left(\omega_{u}^{ul}\left(t-1\right)+\varrho_{u}^{ul}\left(t\right)-r_{ul}^{u}\left(t\right)\right)$$

(10) $$\Omega^{dl}\left(t\right)=\sum_{b\in B}\Omega_{b}^{dl}\left(t\right)=\sum_{b\in B}\sum_{u\in b}\omega_{u}^{dl}\left(t\right)=\sum_{b\in B}\sum_{u\in b}\left(\omega_{u}^{dl}\left(t-1\right)+\varrho_{u}^{dl}\left(t\right)-r_{dl}^{u}\left(t\right)\right)$$

where $\Omega_{b}^{ul}\left(t\right)$, $\Omega_{b}^{dl}\left(t\right)$, $\omega_{u}^{ul}\left(t\right)$ and $\omega_{u}^{dl}\left(t\right)$ are uplink buffer length of BS *b*, downlink buffer length of BS *b*, uplink buffer length of UE *u* and downlink buffer length of UE *u*.

In this research, our goal is to find a D-TDD configuration policy $\pi (\alpha |\left(\Omega^{ul}\left(t\right),\Omega^{dl}\left(t\right)\right)=\left(x,y\right))$ that maximizes the expected discount return of all UE’s sum rate. That is to say, the expected discount return of all UE’s sum rate increases means the UE uplink and downlink data are transmitted in time. In order to achieve this goal, we obtained the utility function $f(t,\left(\Omega^{ul}\left(t\right),\Omega^{dl}\left(t\right)\right)=\left(x,y\right))$ described in Equation (11).

(11) $$\begin{array}{cc} f(t,\left(\Omega^{ul}\left(t\right),\Omega^{dl}\left(t\right)\right)=\left(x,y\right)) & =E[\sum_{\Delta t=0}^{\infty }\left(\gamma^{\Delta t\;mod\;N_{t}}\cdot r_{all}\left(t+\Delta t\right)\right)|\left(\Omega^{ul}\left(t\right),\Omega^{dl}\left(t\right)\right)=\left(x,y\right)] \end{array}$$

where $N_{t}$ is the slot number of a radio frame, and $r_{all}\left(t\right)=\sum_{b\in B}(\left(1-\tau_{b}\left(t\right)\right)\sum_{u\in U}\eta_{b}\left(t\right)r_{ul}^{u}\left(t\right)$ + $\tau_{b}\left(t\right)\sum_{u\in U}\eta_{b}\left(t\right)r_{dl}^{u}\left(t\right))$ represents the sum rate of all UE. Then, D-TDD configuration problem can be formulated as:

(12) $$\begin{array}{cc} \left(P1\right):\min_{\pi (\alpha |\left(\Omega^{ul}\left(t\right),\Omega^{dl}\left(t\right)\right)=\left(x,y\right))} & f(t,\left(\Omega^{ul}\left(t\right),\Omega^{dl}\left(t\right)\right)=\left(x,y\right)))\\ \;s.t.\; & \;C1:\;u\in U,b\in B\\ \; & \;C2:\;\tau_{b}\left(t\right),\eta_{u,b},\kappa_{u,b}^{c}=0or1\\ \; & \;C3:\;\sum_{b\in B}\eta_{u,b}=1,\sum_{b\in B}\sum_{u\in U}\kappa_{u,b}^{c}=1 \end{array}$$

where C1, C2, and C3 are the constraints of indicators, which define that each UE only connects to one BS and sub-channel of each BS cannot reuse. Formulated problem P1 is a dynamic programming problem because of the existence of multiple discrete variables and the nonlinear function form. A dynamic programming problem is NP-hard problem because it combines the difficulty of optimizing over discrete variables with challenges of handling nonlinear functions. Therefore, we are motivated to design an efficient MARL solution.

### 2.2. Proposed Method

In order to reduce control delay and signaling overhead, we deploy a fully distributed MARL algorithm at the edge of the network (each BS) to obtain D-TDD configuration. Each BS learns D-TDD configuration by itself based on local observations under the condition of lacking global information (buffer length, D-TDD configurations, and rates of other BS). This leads to two problems:

- Non-stationary learning environment: The lack of other BS’s buffer length and D-TDD configurations makes the learning environment of each BS non-stationarity. The non-stationarity environment leads to the reward fluctuating greatly under the same local observation and action, which increases the difficulty of learning.
- Relative over-generalization: The lack of other BS rates means that BS prefer to select a sub-optimal action in a joint action space. This results in that a certain BS selects a sub-optimal D-TDD configuration which increases its own data rate but increases interference to other BS. At last, the overall system rate is decreased.

Refer to the experience of applying lenient learning to genetic algorithm, we apply LC-MARL to solve those problem.

In this section, we introduce the proposed LC-MARL based decentralized D-TDD configuration method in three parts. First, we introduce reinforcement model. Second, we explain the principle of LC-MARL. Last, the detail of proposed method is illustrated.

#### 2.2.1. Reinforcement Learning

In reinforcement learning, agents continuously collect states from environment and choose action based on feedback rewards. In our considered scenario, agents are BSs, and actions are candidate D-TDD configurations (UL/DL slots ratio in a frame). After BSs choose the configuration, the network run for the next time frame and feedback of network performance are collected to calculate the rewards of the corresponding actions. We define state, action, and Q-value as follow.

**Figure 2:**
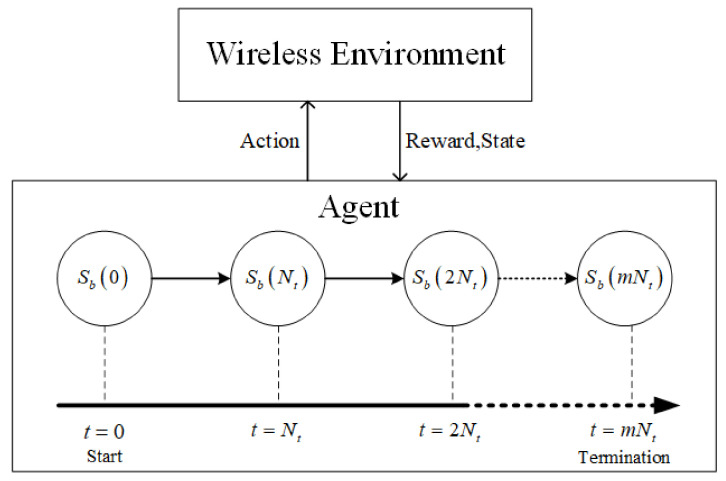
State transition diagram.

1.  State: we format the state $s_{b}^{t}$ of BS *b* at time slot *t* to a tuple, where the element is uplink and downlink buffer length of BS *b*. This is described as:
  (13) $$s_{b}^{t}=\left(\Omega_{b}^{ul}\left(t\right),\Omega_{b}^{dl}\left(t\right)\right)$$
  where $\Omega_{b}^{ul}\left(t\right)$ and $\Omega_{b}^{ul}\left(t\right)$ represents the uplink and downlink buffer length of BS *b* at time slot *t*. We normalize all of the state parameters values. In our proposed method, the scheduling time interval is one frame, that is, $N_{t}$ time slots. Instead of conventional reinforcement learning that only considers the state of one time slot, we consider states of historical time sequence with $N_{t}$ time slots in a frame *t* for representing the state $S_{b}\left(t\right)$ of BS *b* at scheduling moment *t*. Thus, $S_{b}\left(t\right)$ can be formulated as:
  (14) $$S_{b}\left(t\right)=\left(s_{b}^{t-N_{t}},s_{b}^{t-N_{t}+1},\ldots ,s_{b}^{t-1}\right)$$
  where the number of $N_{t}$ is obtained by Table 1 according to numerology μ. BS *b* performs D-TDD configuration at scheduling moment *t*. After the $N_{t}$ time slots, BS obtains the next state $S_{b}\left(t+N_{t}\right)$ feedback by the wireless environment. Then, BS *b* performs the configuration at the scheduling moment $t_{N_{t}}$. The process of state transition is described in Figure 2, where the termination condition is that all UE buffers are cleared.
2.  Action: in D-TDD configuration task, the action space *W* of each BS is the available configurations of UL/DL ratio. According to [26], we consider that there are six different types of duplex pattern. Thus, the action of BS *b* at time *t* is defined as
  (15) $$\begin{array}{cc} \alpha_{b}\left(t\right) & =(N_{up},N_{down})\in W\\ W & =\left\{(1,9),(2,8),(3,7),(4,6),(5,5),(6,4)\right\} \end{array}$$
  where $N_{up}$ and $N_{down}$ are uplink and downlink ratio. BS selects action following ϵ−greedy policy according to Q-value of tuple $\left(S_{b}\left(t\right),\alpha_{b}\left(t\right)\right)$, and the calculation method of Q-value is described in the next part.
3.  Q-value: after agent selects action, the reward $R_{b}\left(t\right)$ is calculated to evaluate the feedback of tuple $\left(S_{b}\left(t\right),\alpha_{b}\left(t\right)\right)$ and agent transits to the next state $S_{b}\left(t+N_{t}\right)$. As described in Equation (11), our purpose is to transmit uplink an downlink services data in time. Therefore, the reward function of tuple $\left(S_{b}\left(t\right),\alpha_{b}\left(t\right)\right)$ can be defined as:
  (16) $$R_{b}\left(t\right)=-\left(r_{b}^{up}\left(t\right)+r_{b}^{down}\left(t\right)\right)=-\left(\sum_{u\in b}\omega_{u}^{ul}\left(t+N_{t}\right)+\sum_{u\in b}\omega_{u}^{dl}\left(t+N_{t}\right)\right)$$
  In the Formula (16), $r_{b}^{ul}\left(t\right)$ and $r_{b}^{dl}\left(t\right)$ denote the uplink and downlink reward at time *t*, which are negatively correlated with the sum of intra-BS UE uplink buffer length $\omega_{u}^{ul}\left(t+N_{t}\right)$ and downlink buffer length $\omega_{u}^{dl}\left(t+N_{t}\right)$ at the beginning of the next scheduling moment $t+N_{t}$. In terms of Bellman Equations for deterministic policies [27], the Q-value of tuple $\left(S_{b}\left(t\right),\alpha_{b}\left(t\right)\right)$ can be written as:
  (17) $$Q\left(S_{b}\left(t\right),\alpha_{b}\left(t\right)\right)=R_{b}\left(t\right)+\gamma \max_{\alpha \in W}Q\left(S_{b}\left(t+N_{t}\right),\alpha_{b}\left(t+N_{t}\right)\right)$$
  For high dimensions of states, it is difficult to maintain a Q-table for agents. So we use a neural network (NN) to estimate the Q-value. Agents save $(S_{b}\left(t\right),\alpha_{b}\left(t\right),S_{b}\left(t+N_{t}\right),R\left(S_{b}\left(t\right),\alpha_{b}\left(t\right)\right)$ of each exploration into experience reply memory (ERM). During training, agents select a training mini-batch from ERM to train the online NN. The benchmark value $Y(S_{b}\left(t\right),\alpha_{b}\left(t\right))$ for online NN is calculated by
  (18) $$Y\left(S_{b}\left(t\right),\alpha_{b}\left(t\right)\right)=R_{b}\left(t\right)+\gamma Q^{*}\left(\phi \left(S_{b}\left(t+N_{t}\right)\right),\max_{\alpha \in W}Q\left(\phi \left(S_{b}\left(t+N_{t}\right)\right),\alpha_{b}\left(t+N_{t}\right),\theta \right),\theta^{*}\right)$$
  where θ is the hyper-parameter vector of neural network, $\phi (S_{b}\left(t+N_{t}\right))$ is the features extracted by LSTM of the next time series state. $Q^{*}$ means the Q-value calculated by another NN called target NN. In the process of benchmark value calculation, online NN is used for selecting the action $\alpha_{b}\left(t+N_{t}\right)$ of the next state $S_{b}\left(t+N_{t}\right)$, and target NN is used for estimating the Q-value of the next state tuple $\left(S_{b}\left(t+N_{t}\right),\alpha_{b}\left(t+N_{t}\right)\right)$. Every *G* steps, the hyper-parameter of online NN is copied to target NN. This method prevents Q-value over-estimation issue [28,29].

In distributed MARL, each BS is an agent that independently completes a learning task based on their local observation, which saves the resources used by data transmission. However, that would cause the problem of no-stationary environment and relative over-generalization. So, we apply the LC-MARL algorithm to solve this problem. In the following part, we introduce the LC-MARL in terms of leniency control process and auto-encoder based hashing process.

#### 2.2.2. Leniency Control Based Distributed MARL

The idea of leniency control is derived from the application of lenient learning in [30] by Potter and De Jong, they applied lenient learning to extend traditional GA which improves the performance of solving complex problems. Ref. [31,32] applied lenient learning to MARL. In [31], the author verified that leniency control can effectively prevent relative over-generalization. The motivation of [32] is to help co-evolutionary algorithms converge towards a sub-optimal policy, which is a difficult task because the noise generated by other agent’s exploration strategy makes learning environment non-stationary. When we use distributed MARL to solve the D-TDD configuration problem, the noise is caused by other BS’s D-TDD configuration strategy. So, we apply a leniency control to solve the problem of non-stationary learning environment in D-TDD configuration tasks. As described in Equation (19), leniency controller maintains a leniency value for each tuple $\left(S_{b}\left(t\right),\alpha_{b}\left(t\right)\right)$.

(19) $$l\left(S_{b}\left(t\right),\alpha_{b}\left(t\right)\right)=1-e^{-K*T(S_{b}\left(t\right),\alpha_{b}\left(t\right))}$$

where *K* is a leniency moderation factor, and $T(S_{b}\left(t\right),\alpha_{b}\left(t\right))$ is temperature function. Each state–action pair initially assigned a defined maximum temperature value, and temperature value $T(S_{b}\left(t\right),\alpha_{b}\left(t\right))$ decayed each time that the state–action pair is visited. Following the update, $T_{t}\left(S_{b}\left(t\right),\alpha_{b}\left(t\right)\right)$ is decayed using a discount factor β∈[0,1], such that $T_{t+1}\left(S_{b}\left(t\right),\alpha_{b}\left(t\right)\right)=\beta T_{t}\left(S_{b}\left(t\right),\alpha_{b}\left(t\right)\right)$. Thus, we can find that leniency value of a state–action pair decreases when the times it is visited increases. Given a TD-Error δ, where $\delta =Y\left(S_{b}\left(t\right),\alpha_{b}\left(t\right)\right)-Q\left(S_{b}\left(t\right),\alpha_{b}\left(t\right),\theta \right)$, leniency control is applied to the calculation of benchmark value as follows:

(20) $$Y_{l}\left(S_{b}\left(t\right),\alpha_{b}\left(t\right)\right)=\left\{\begin{array}{cc} Q(S_{b}\left(t\right),\alpha_{b}\left(t\right),\theta )+\delta & \delta >0\;or\;x>l(S_{b}\left(t\right),\alpha_{b}\left(t\right))\\ Q(S_{b}\left(t\right),\alpha_{b}\left(t\right),\theta ) & \delta \leq 0\;and\;x\leq l(S_{b}\left(t\right),\alpha_{b}\left(t\right)) \end{array}\right.$$

**Figure 3:**
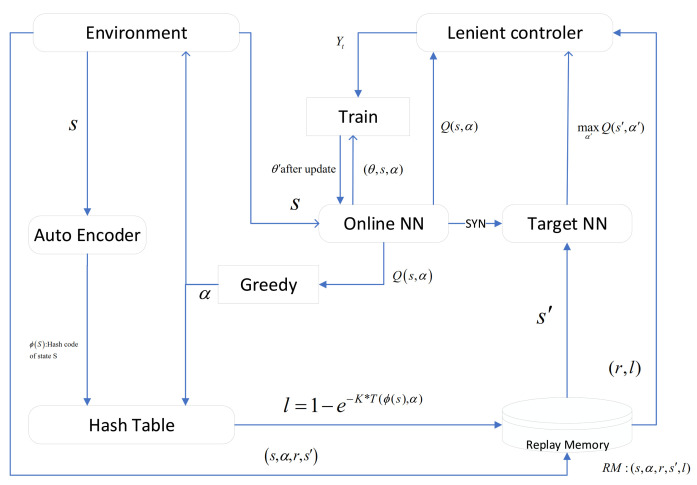
Process of LC-MARL.

The random variable x∼U(0,1) is adopted to ensure that an update on a negative δ is executed with a probability $1-l(S_{b}\left(t\right),\alpha_{b}\left(t\right))$. As shown in Equation (20), when the TD-error of the Q-value is less than 0, the lenient controller tends to ignore the sampling of this action. This reduces the impact of noise on learning in a non-stationary learning environment. On the other hand, the lenient controller does not ignore all samples when TD-error is less than 0, but effectively ignores it with probability $1-l(S_{b}\left(t\right),\alpha_{b}\left(t\right))$. According to Equations (19) and (20), leniency control tends to forgive (ignore) sub-optimal Q-value iteration in the initial exploration phase. As the agent explores, the probability of leniency control ignoring the sub-optimal Q-value decreases. That is to say, an agent is less lenient to a frequently visited state–action tuple Q-value iteration, while quite lenient in unexplored areas. This also avoids agents iterating towards a local optimum instead of a global optimum, that is, a relative over-generalization. Thus, leniency control increases the ability of convergence towards the globally optimal solution in distributed MARL. The specific process of LC-MARL is shown in Figure 3.

LC-MARL fulfill decentralized D-TDD configuration task, but it has a shortcoming: weak learning ability in a high-dimensional space environment. In D-TDD configuration learning task, the state is defined as a time series of the UE buffer, whose space dimension is high due to that the number of slots in a frame is large. So, maintaining a leniency value of each visited state–action pair is no longer feasible. Motivated by this shortcoming, we apply a binary auto-encoder based self-supervised temporal hashing method to improves the learning ability of LC-MARL in a high-dimensional environment.

#### 2.2.3. Binary Auto-Encoder Based Self-Supervised Temporal Hashing

**Figure 4:**
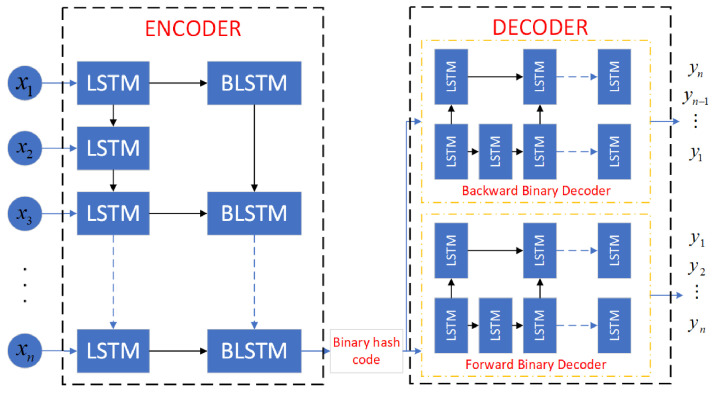
Binary auto-encoder.

Recently, many studies focus on the application of auto-encoder to reinforcement learning. For example, auto-encoders were adopted to automatically cluster states in a meaningful way in Montezuma’s Revenge [33]. So, we apply binary auto-encoder to reduce the dimension of $S_{b}\left(t\right)$. As shown in Figure 4 binary auto-encoder consists of an encoder layer and a decoder layer which contains two decoders named backward binary decoder and forward binary decoder, respectively.

Encoder layer extracts the feature of inputs and output a lower dimension binary code, then decoder layer reconstructs the inputs from binary code. The loss function of auto-encoder is the mean squared error between decoder layer’s outputs and inputs. After training, the encoder layer of binary auto-encoder serves as a temporal hashing function $\Phi \left(S_{b}\left(t\right)\right)\in {\left\{1,-1\right\}}^{k})$, which reduce the dimension of state to *k*. So, we use hash-key output by $\Phi (S_{b}\left(t\right))$ to maintain leniency value of state–action pair, which can be rewritten as

(21) $$l\left(S_{b}\left(t\right),\alpha_{b}\left(t\right)\right)=1-e^{-K*T(\Phi \left(S_{b}\left(t\right)\right),\alpha_{b}\left(t\right))}$$

**Figure 5:**
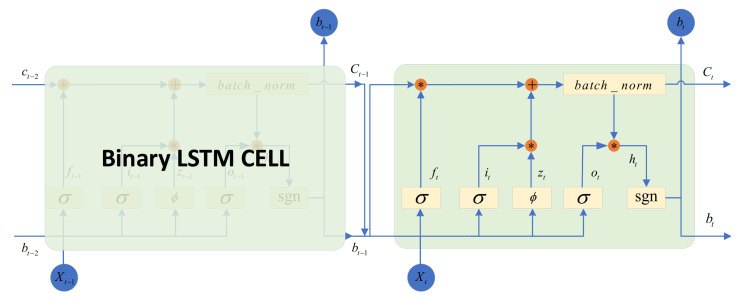
Binary LSTM.

Different from [33], state is defined as time series in D-TDD configuration task. So as illustrated in Figure 5, a novel variant of LSTM named BLSTM is adopted to generate a binary hash code in encoder layer.

The traditional method of extracting binary features of time series is to add a binary output layer to the LSTM-based feature extraction layer. This method is more likely to lose information in the time series. Therefore, we binarize the hidden state $h_{t}$ of LSTM and add it to recurrent calculation process. At the same time, in order to output effective binary information and avoid gradient explosion, we added a batch normalization layer to output normalized LSTM cell state information $c_{t}$. The detailed implementation of BLSTM is given as follows:

(22) $$\begin{array}{cc} \; & f_{t}=\sigma \left(W_{xf}x_{t}+U_{bf}b_{t-1}+M_{cf}\circ c_{t-1}+b_{f}\right) \end{array}$$

(23) $$\begin{array}{cc} \; & i_{t}=\sigma \left(W_{xi}x_{t}+U_{bi}b_{t-1}+M_{ci}\circ c_{t-1}+b_{i}\right) \end{array}$$

(24) $$\begin{array}{cc} \; & o_{t}=\sigma \left(W_{xo}x_{t}+U_{bo}b_{t-1}+M_{co}\circ c_{t-1}+b_{o}\right) \end{array}$$

(25) $$\begin{array}{cc} \; & z_{t}=\phi \left(W_{xm}x_{t}+U_{bm}\circ b_{t-1}+b_{m}\right) \end{array}$$

(26) $$\begin{array}{cc} \; & c_{t}=batch\_norm\left(f_{t}\circ c_{t-1}+i_{t}\circ z_{t}\right) \end{array}$$

(27) $$\begin{array}{cc} \; & h_{t}=o_{t}\circ c_{t} \end{array}$$

(28) $$\begin{array}{cc} \; & b_{t}=sgn\left(h_{t}\right) \end{array}$$

where ∘, σ and ϕ denotes the element-wise multiplication, sigmoid function, and tanh function.

#### 2.2.4. Details of Proposed Method

We can find the process of proposed method as shown in Algorithm 1. First, we initialize ERM, online NN, and target NN. Then, the algorithm enters LC-MARL training stage. The training phase contains M episodes. In each episode, agents learn by continuously exploring environment until it satisfies termination condition. In D-TDD configuration task, the termination condition is that all UE buffers are cleared or exploration step reaches the maximum value. In each step, each BS uses online NN to estimate Q-value of all available duplex patterns under local observation $S_{b}\left(t\right)$, and then selects the duplex patterns $\alpha_{b}\left(t\right)$ according to ϵ-greedy strategy. After $N_{t}$ time slots, the wireless network environment feeds back the reward of each BS. Leniency controller of each agent calculates the leniency value of state–action pair $\left(S_{b}\left(t\right),\alpha_{b}\left(t\right)\right)$ in this step, and stores the data of this step into ERM. Finally, each agent extracts a mini-batch from the ERM to train the online network, and synchronizes the online NN and target NN every G step.
**Algorithm 1** LC-MARL based decentralized D-TDD configuration1:Initialize the wireless network environment2:Initialize the ERM of each BS3:Initialize the online NN of each BS with weight $\theta_{b}$4:Initialize the target NN of each BS with weight $\theta_{b}^{*}=\theta_{b}$5:/* Traning */6:**for** episode=1,2,⋯,M **do**7:    Initialize uplink and downlink buffer length of each UE8:    t=09:    **while** the termination condition is not satisfied **do**10:        /*Action Seclect*/11:        **for** BS *b* in *B* **do**12:           Observe local state $S_{b}\left(t\right)$13:           Use online NN to estimate Q-value of all $\left(S_{b}\left(t\right),\alpha_{b}\right),\forall \alpha_{b}\in W$14:           Choose duplex pattern $\alpha_{b}\left(t\right)={\mathrm{argmax}}_{\alpha_{b}\in W}Q\left(S_{b}\left(t\right),\alpha_{b}\right)$ with probability 1−ϵ15:        **end for**16:        Global duplex pattern $\alpha \left(t\right)=[\alpha_{1}\left(t\right),\ldots ,\alpha_{N_{bs}}\left(t\right)]$17:        Feed global duplex pattern to wireless communication environment18:        Calculate the SINR of each UE *u* according to Equations (3) and (4)19:        Calculate reward $R_{b}\left(t\right)$ and evolved state $S_{b}\left(t+N_{t}\right)$ of each BS *b*20:        /*Online NN Training*/21:        **for** BS *b* in *B* **do**22:           Calculate leniency value $l(S_{b}\left(t\right),\alpha_{b}\left(t\right))$ according to Equation (21)23:           Store experience tuple $\left(S_{b}\left(t\right),\alpha_{b}\left(t\right),R_{b}\left(t\right),S_{b}\left(t+N_{t}\right),l\left(S_{b}\left(t\right),\alpha_{b}\left(t\right)\right)\right)$ to ERM24:           Sample a mini-batch of ERM25:           Calculate benchmark value according to Equation (20)26:           Perform gradient descent to train online NN of BS *b*27:        **end for**28:        /* parameter synchronization */29:        Every *G* steps, set $\theta_{b}^{*}=\theta_{b}$30:        $t=t+N_{t}$31:    **end while**32:**end for**33:Output trained online NN of each BS34:Obtain D-TDD configuration of each BS by Formula (29)35:Obtain global D-TDD configuration by Formula (30)

After training process, we obtain each BS’s D-TDD configuration policy $\pi_{b}\left(\alpha_{b}|S_{b}\left(t\right)\right)$ by trained online NN of each BS, as shown in Equation (29).

(29) $$\begin{array}{c} \pi_{b}\left(\alpha_{b}|S_{b}\left(t\right)\right)\leftarrow \left\{\begin{array}{cc} 1 & if\;\alpha =A^{*},A^{*}=argmax_{\alpha \in W}Q\left(\phi \left(S_{b}\left(t\right)\right),\alpha_{b}\left(t\right),\theta \right)\\ 0 & else \end{array}\right. \end{array}$$

Because each BS obtains its own D-TDD configuration through independent training, we obtain the global D-TDD configuration policy π(α|S(t)) by Formula (30).

(30) $$\begin{array}{cc} \pi (\alpha |S(t)) & =\prod_{b\in B}\pi_{b}\left(\alpha_{b}|S_{b}\left(t\right)\right)\\ \alpha & =[\alpha_{1},\alpha_{2},\ldots \alpha_{N_{bs}}]\\ S(t) & =[S_{1}\left(t\right),S_{2}\left(t\right),\ldots S_{N_{bs}\left(t\right)}] \end{array}$$

It is worth mentioning that because LC-MARL independently learns D-TDD configuration strategy of each BS, it does not require UE service information of other BSs, which reduces signaling overhead and avoids data transmission between BSs. At the same time, our proposed method can be deployed on the MEC. Compared with centralized method, our proposed method is located closer to wireless network, which reduces the time for transmitting D-TDD configuration decisions to wireless network.

## 3. Results

In this section, we carry out a simulation to validate our proposed lenient-MARL based D-TDD duplex control framework. First, we investigate the convergence of our proposed MARL algorithms. Second, we evaluate the performance of the framework in terms of overall system rate and each BS data transmission rate. For the simulation environment, we set up a server powered by a dual Intel(R) Xeon CPU 2.40 GHz 4-cores and 16 GB RAM. We use python3.8 for the wireless communication simulation. Further, we build BLSTM, auto-encoder, and deep learning static graph with tensorflow1.0.

A summary of the wireless environment simulation parameters is provide in Table 3. We assumed that each BS uses the same frequency. All UEs are distributed within BS coverage area, and the number of UEs in the coverage area follow Poisson distribution with expectation $\lambda_{UE}$. Each UE selects BS which provides the max reference signal received power RSRP as a serving BS. $\lambda_{h}$ and $\lambda_{l}$ represent the high-load and low-load packet generation rates, respectively. The UE uplink and downlink high-load probability is represented by $p_{UL}^{high}$ and $p_{DL}^{high}$. Moreover, four types of traffic are considered in our simulation, namely high uplink load, high downlink load, high load, and low load. The specific parameters of the four flow types are shown in Table 4.

Table 5 summarizes the hyper-parameters of LC-MARL. In order to estimate Q-value of time series state, we extract feature of the time series state through the LSTM layer. Then, we construct a three-layer fully connected neural network, with the number of cells in each layer decreasing. For each learning step, *Adam* is adopted to optimize the hyper-parameter of online NN. This method computes individual adaptive learning rates for different parameters from estimates of first and second moments of the gradients [34], and the initial learning rate is shown in Table 5. The discount rate is 0.9. A higher discount rate value enhances the effect of future reward on Q-value and encourages agents to learn a policy to reach the termination faster. In this case, that means the data in the buffer are transmitted in time.

**Figure 6:**
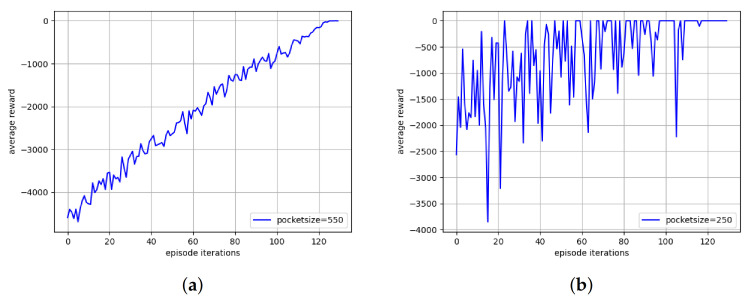
Convergence of LC-MARL.(**a**) packet size-550; (**b**) packet size-250.

Figure 6a,b plot the convergence of our proposed LC-MARL algorithm with different packet size. In the figure, the *x*-axis and *y*-axis represents episode steps and total system reward. It is clear that total system reward increases with continuous training. Unlike Figure 6a,b has a larger total reward jitter.

For network performance analysis, we compare the proposed LC-MARL solution with decentralized deep reinforcement learning (DDRL) in terms of overall system rate and rate of distribute agents (each BS). For the detail of DDRL, we expand deep reinforcement learning proposed by paper [26] to multi-agent domain, which is a common learning cooperation MARL solution [35]. In DDRL based method, each BS obtains the D-TDD configuration through parallel distributed learning. At each scheduling moment, BS performs a D-TDD configuration based on state information of wireless environment, and obtains reward and next state feedback from environment at next scheduling moment, then stores data in memory. At each scheduling moment, BS trains a NN to estimate the Q-value through memory replay method. Compared with our proposed method, DDRL based method does not use auto-encoder based leniency control to avoid non-stationary learning environment issue and relative over-generalization issue in MARL.

**Figure 7:**
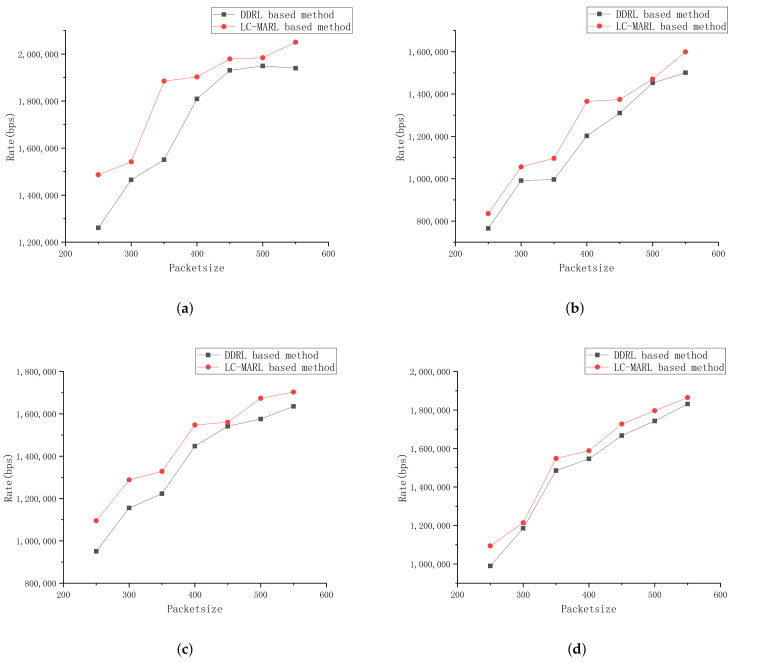
Overall rate comparison in different traffic type. (**a**) high load; (**b**) low load; (**c**) high uplink load; (**d**) high downlink load.

Figure 7 shows the overall system rate of different traffic types with 4 SBs, where overall system rate is the sum of each BS’s UL/DL transmission rate. In the figure, the *x*-axis and *y*-axis represents packet size and sum rate of all UEs. As shown in the figure, compared with DDRL based framework, our proposed method can reach a higher overall system rate under all four traffic types.

To verify the performance of our proposed algorithm under different numbers of BSs, we carried out simulations where the number of BSs is 2, 3, 4, and 5. The simulation results are shown in Table 6, where $Rate_{DDRL}$ and $Rate_{LC-MARL}$ are the overall system rate of DDRL based method and the overall system rate of LC-MARL based method. We reflect the advantages of the proposed method through performance gain which is calculated by the overall system rate of LC-MARL based method minus the overall system rate of DDRL based method. As shown in Table 6, as the number of agents increases, the performance gains of our proposed method are higher.

**Figure 8:**
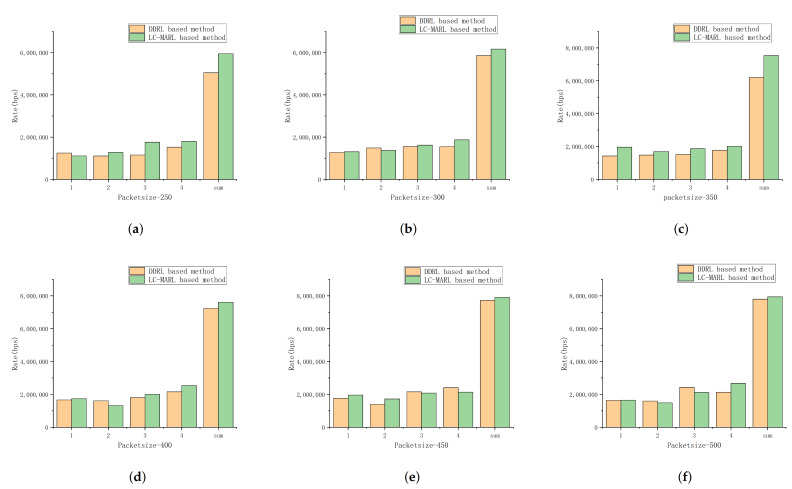
BS rate and overall rate comparison in different packet size.(**a**) packet size-250; (**b**) packet size-300; (**c**) packet size-350; (**d**) packet size-400; (**e**) packet size-450; (**f**) packet size-500.

We further evaluate the performance of our proposed framework by comparing the distributed benefits of our framework and DDRL based solution. Figure 8 describes the data rate of each BS and overall system rate under different packet sizes. It can be seen from the figure that our proposed framework can achieve a higher overall system rate through distributed learning. Taking Figure 8d as an example, compared with our solution, the DDRL based solution increases the data transmission rate of BS 2, but our solution increases the overall system rate.

## 4. Discussion

The curve in Figure 6b converges faster and has greater jitter. This is because the packet size of simulation in Figure 6b is smaller than that in Figure 6a. For LC-MARL, the D-TDD configuration policy is easier to learn with less network load. In D-TDD configuration task, the termination condition of each episode is for all UE buffer to be cleared. So, in each training episode, agent reaches the end of episode through fewer training steps, which reduces the number of samples in each episode. Therefore, total system reward of each episode jitter greatly. However, it is observed that total system reward gradually stabilizes as the training progresses and converges at 110 episodes. In other words, the algorithm completes exploration of scenarios with less load through less training.

As shown in Figure 7, compared to DDRL based framework our proposed control framework performance well on overall system rate in all traffic types. This is because DDRL based scheme is a fully decentralized MARL method, where the learning goal of each BS is to improve service quality of BS rather than overall system rate. Although our solution has the same learning goals as the DDRL based solution, we improve overall benefits of fully distributed MARL method through proposed effective leniency control. In addition, the overall system rate under high load traffic increases slowly, because the system rate gradually reaches the limit of system capacity as the size of the data packet increases under high load traffic.

As described in Figure 8, the DDRL based method is more likely to fall into local optimum for the absence of effective global information. This is because a sub-optimal policy in joint action space is preferred over an optimal policy for the distributed agents in DDRL based method. This is the relative over-generalization issue in MARL. Compared to DDRL based method our proposed method improves overall system rate by effectively controlling the calculation process of the target Q-value according to Equation (20). Furthermore, auto-encoder improve the ability of leniency control to handle the high dimensional learning environment in D-TDD configuration task. This also enhances the performance of our proposed method. Moreover, as the number of BSs increases, the dimension of the joint action space becomes higher. This leads to a more serious problem of relative over-generalization. Therefore, the performance gain of the proposed method in Table 6 increases with the number of BSs increasing.

## 5. Conclusions

In this paper, we developed a D-TDD framework for 5G NR that allows each BS dynamic adjust duplex pattern to adapt services buffer. In order to reduce signaling overhead and control delay, we designed a distribute MARL based decentralized D-TDD configuration solution. Further, our proposed LC-MARL based D-TDD configuration method uses leniency control to enable BSs cooperate configure duplex pattern based on local observations. LC-MARL is a learning cooperation method to improve MARL performance. Different from learning communication method, learning cooperation means agent maximizes their shared reward without communication. This is the reason why LC-MARL can reduce signaling overhead and control delay. In order to verify the performance of our proposed method, we perform our proposed method performance simulations in different scenarios and compare them with another learning cooperation MARL method. Simulation showed that the proposed MARL converged stably in various environments. Compared with another learning cooperation method (DDRL), our solution provides data rate gains. For our future work, we will continue to consider other available duplex method in B5G technology.

## Figures and Tables

**Table 3:** Simulation parameters of wireless environment.

Parameter	Value
BS number	2, 3, 4, 5
Sub-channel number	10
Sub-channel bandwidth	1 Mhz
BS power	46 dbm
BS distance	500 m
$\lambda_{UE}$	8
$\lambda_{h}$	325
$\lambda_{l}$	125

**Table 4:** Traffic type.

Traffic Type	$p_{\mathrm{UL}}^{\mathrm{high}}$	$p_{\mathrm{DL}}^{\mathrm{high}}$
High uplink load	0.8	0.2
High downlink load	0.2	0.8
High load	0.8	0.8
Low load	0.2	0.2

**Table 5:** Hyper-parameters of LC-MARL.

Component	Hyper-Parameter	Setting
Reinforcement Learning	Learning rate	0.005
	Discount rate	0.9
	Synchronization steps	30
	Memory size	1500
	Batch size	100
	Maximum step in a episode	500
	Cell number	20
	LSTM hidden state number	5
	Hidden layer number	3
ϵ-greedy	Initial value	1
	Minimum value	0.05
Leniency control	MaxTemperatur	1
	Temperature modification coefficient K	2
	Initial max temperature rate	1
Auto-encoder	Max temperature decay coefficient	0.999
	Hash-key dimensions	5
	Binary LSTM hidden state number	5
	LSTM layer number	2

**Table 6:** Performance comparison in different BS numbers.

BS Number	${\mathrm{Rate}}_{\mathrm{DDRL}}$ (bps)	${\mathrm{Rate}}_{\mathrm{LC}-\mathrm{MARL}}$ (bps)	Performance Gain
2	896,956	932,579	35,623
3	1,003,520	1,031,180	27,660
4	1,174,080	1,237,370	63,290
5	1,193,120	1,281,630	88,510

## Data Availability

Not applicable.

## Abbreviations

The following abbreviations are used in this paper:

3GPP	3rd generation partnership project
5G	5th generation mobile communication technology
NR	New radio
B5G	Beyond 5G
URLLC	Ultra-reliable and low latency communications
IoT	Internet of things
IoV	Internet of vehicle
D-TDD	Dynamic time division duplex
S-TDD	static time division duplex
LTE	Long-term evolution
TD-LTE	Time division long-term evaluation
MARL	Multi-agent deep reinforcement learning
BS	Base station
UE	User
LSTM	Long short-term memory
BLSTM	Binary LSTM
MEC	Mobile edge computing
UL	Uplink
DL	Downlink
LC-MARL	Leniency control based MARL
DDRL	Decentralized deep reinforcement learning
OFDM	Orthogonal frequency-division multiplexing
NN	Neural network
*U*	The set of UE
*B*	The set of BS
*C*	The set of reused orthogonal sub-channels of each BS
μ	Numerology parameter
δ	UL/DL transmission periodicity
$\eta_{u,b}$	Association indicator of UE *u* between BS *b*
$\tau_{b}\left(t\right)$	Transmission direction of BS *b* at time *t*
$\kappa_{u,b}^{c}$	Association indicator of UE *u* between BS *b* on sub-channel *c*
$\gamma_{u}^{ul}\left(t\right)$	Uplink SINR of UE *u* at time *t*
$\gamma_{u}^{dl}\left(t\right)$	Downlink SINR of UE *u* at time *t*
$r_{ul}^{u}\left(t\right)$	Uplink rate of UE *u*
$r_{dl}^{u}\left(t\right)$	Downlink rate of UE *u*
$\Omega_{b}^{ul}\left(t\right)$	Uplink buffer length of BS *b*
$\Omega_{b}^{dl}\left(t\right)$	Downlink buffer length of BS *b*
$\omega_{u}^{ul}\left(t\right)$	Uplink buffer length of UE *u*
$\omega_{u}^{dl}\left(t\right)$	Downlink buffer length of UE *u*
$s_{b}^{t}$	State of BS *b* at time *t*
$S_{b}^{t}$	Time series state of BS *b* at time *t*
$\alpha_{b}\left(t\right)$	Action of BS *b* at time *t*
$R_{b}\left(t\right)$	Reward of BS *b* fed by environment at time *t*
γ	Discount factor
$Q(S_{b}\left(t\right),\alpha_{b}\left(t\right))$	Q-value of tuple $\left(S_{b}\left(t\right),\alpha_{b}\left(t\right)\right)$
$Q(S_{b}\left(t\right),\alpha_{b}\left(t\right),\theta )$	Q-value calculated by online NN
$Q^{*}\left(S_{b}\left(t\right),\alpha_{b}\left(t\right),\theta \right)$	Q-value calculated by target NN
θ	Hyper-parameter of online NN
θ	Hyper-parameter of target NN
$Y(S_{b}\left(t\right),\alpha_{b}\left(t\right))$	Benchmark value of tuple $\left(S_{b}\left(t\right),\alpha_{b}\left(t\right)\right)$
$l(S_{b}\left(t\right),\alpha_{b}\left(t\right))$	Leniency value of tuple $\left(S_{b}\left(t\right),\alpha_{b}\left(t\right)\right)$
*K*	Leniency moderation factor
$T(S_{b}\left(t\right),\alpha_{b}\left(t\right))$	Temperature function of tuple $\left(S_{b}\left(t\right),\alpha_{b}\left(t\right)\right)$
δ	TD-Error
$\pi_{b}\left(\alpha_{b}|S_{b}\left(t\right)\right)$	D-TDD configuration policy of BS *b*
π(α|S(t))	Global D-TDD configuration policy
